# Tropical Islands as New Hubs for Emerging Arboviruses

**DOI:** 10.3201/eid2205.150547

**Published:** 2016-05

**Authors:** Van-Mai Cao-Lormeau

**Affiliations:** Institut Louis Malardé, Papeete, Tahiti, French Polynesia

**Keywords:** chikungunya, Zika, dengue, Indian Ocean, Pacific, Caribbean, tropical, islands, arboviruses, vector-borne infections, viruses

**To the Editor:** The outbreaks of dengue virus (DENV), chikungunya virus (CHIKV), and Zika virus infection that occurred on islands in the Indian Ocean, the Pacific, and the Caribbean over the past decade have demonstrated the potential of these arboviruses to pose a global public health threat. All 3 viruses were first isolated in the mid-20th century in either African or Asian countries; however, until 2005, only DENV (family *Flaviviridae*, genus *Flavivirus*) was considered a global public health concern (*1*).

In 2005, CHIKV infection, which typically manifests as fever, joint pain, rash, and polyarthralgia, emerged on islands in the Indian Ocean. During the next 10 years, CHIKV (family *Togaviridae*, genus *Alphavivirus*) caused several outbreaks in the Indian subcontinent, Asia, and Central Africa, and autochthonous transmission was reported in Europe (*2*). In 2011, CHIKV appeared for the first time in the Pacific region; 2 years later, it had expanded throughout the region (*3*). At the end of 2013, CHIKV emerged in the Caribbean and subsequently spread to the continental Americas, resulting in 1,726,539 suspected and 60,746 laboratory-confirmed CHIKV infections in the region as of December 18, 2015 (http://www.paho.org/hq/index.php?option=com_docman&task=doc_download&Itemid=&gid=30198&lang=en).

In 2007, Zika virus (family *Flaviviridae*, genus *Flavivirus*), which typically manifests as fever, joint pain, rash, and conjunctivitis, emerged for the first time outside Africa and Asia, in Yap State in Micronesia. Six years later, the virus caused a large outbreak in French Polynesia and then spread to other Pacific islands (*3*). In May 2015, autochthonous cases of Zika virus infection were confirmed in Brazil. By the end of the year, Brazil had declared an outbreak, and the virus had spread to several neighboring countries (http://www.paho.org/hq/index.php?option=com_docman&task=doc_download&Itemid=&gid=30198&lang=en).

The emergence of CHIKV and Zika virus in the Indian Ocean, the Pacific, and the Caribbean might result from multiple drivers. One factor is the presence of competent vectors, including the widely distributed *Aedes aegypti* and *Ae.* *albopictus* mosquitoes, but also endemic *Aedes* species that might serve as additional vectors, such as *Ae. hensilli* mosquitoes in Yap State (*4*). Small tropical islands also offer contexts conducive to mosquito proliferation and disease transmission; most meet the criteria to be listed as Small Islands Developing States and territories (SIDS) in the United Nations’ framework of programs of action for sustainable development (http://www.un.org/documents/ga/conf167/aconf167-9.htm). SIDS are characterized by environments that are particularly sensitive and prone to natural disasters, populations that often lack safe water supplies and sanitation, and local governments that have limited resources to implement vector control and manage outbreaks. The increasing volume of travel between SIDS and continental regions where CHIKV and Zika virus are endemic has facilitated the spread of these viruses to previously unexposed populations.

Recent outbreaks of chikungunya and Zika have led to unexpected observations regarding the virulence and epidemic potential of such viruses. The occurrence of severe clinical symptoms in CHIKV infection (e.g., persistent arthralgia, destructive arthritis, and fulminant hepatitis) were documented by Renault et al. (*5*) during an outbreak in La Réunion Island during 2005–2006 (*2*). The severity of the outbreaks in the Indian Ocean was further correlated with the occurrence of specific mutations in the CHIKV genome that enabled highly efficient transmission of the mutated Indian Ocean lineage by *Ae. albopictus* mosquitoes (*2*,*4*). Later, chronic polyarthralgia and CHIKV infection–related deaths, most in the elderly and patients with co-morbid conditions, were reported in the Caribbean and the Pacific regions during outbreaks caused by the CHIKV Asian genotype (*4*). Zika-related neurologic disorders and a 20-fold increase in the incidence of Guillain-Barré syndrome were first reported during the outbreak in French Polynesia during 2013–2014. Cases of Guillain-Barré syndrome were also recorded during the Zika outbreak in Brazil (*6*). Moreover, soon after health authorities in Brazil warned of an increase in the prevalence of microcephaly in newborns that might be associated with Zika virus infection in mothers during pregnancy, health authorities in French Polynesia confirmed that neurologic congenital abnormalities also had been observed during the Zika outbreak there (*6*).

Other lessons learned from the emergence of CHIKV and Zika virus in small tropical islands include evidence of nonvectorborne virus transmission and its associated public health implications. Perinatal transmission of Zika virus to a neonate was first described in infected pregnant women in French Polynesia, and possible transplacental transmission was further corroborated by the detection of the virus in amniotic fluid samples of 2 pregnant women in Brazil whose fetuses had been diagnosed with microcephaly (*6*). Sexual transmission of Zika virus, suggested by Foy et al. (*7*), was corroborated by detection of virus in the semen of a patient in French Polynesia (*8*). To prevent transmission of CHIKV and Zika virus by blood transfusion, local blood banks in French Polynesia and the Caribbean adjusted their algorithms for blood donation and screening of blood products during outbreaks (*9*,*10*).

When we observe the geographic distribution of DENV, CHIKV, and Zika virus over the past decade, DENV expansion appears to have been a continuous process. However, the emergence of CHIKV, first in the Indian Ocean and later in the Caribbean, and the emergence of Zika virus in the Pacific has dramatically expanded the reach of these viruses (Figure).

**Figure:**
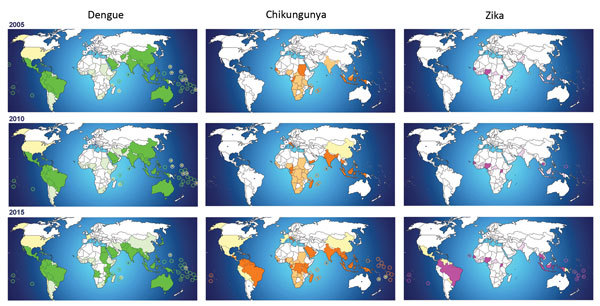
Areas affected by dengue, chikungunya, and Zika viruses, worldwide, 2005, 2010, and 2015, illustrating the evolution of the geographic distribution of these viruses over the past decade (*1*–*5*,*7*). Light shading/circles indicate countries with endemic transmission; dark shading/circles indicate countries with outbreaks recorded during the previous 5 years; dots indicate imported cases in countries without autochthonous transmission; stars indicate countries with reported autochthonous transmission.
